# Posterior tibial artery pseudoaneurysm: a rare complication following orthopedic surgery—a case report

**DOI:** 10.1093/jscr/rjaf288

**Published:** 2025-05-07

**Authors:** Estelle Bodart

**Affiliations:** Department of Thoracic and Vascular Surgery, Clinique Saint-Pierre, Ottignies 1340, Belgium

**Keywords:** posterior tibial artery, arterial pseudoaneurysm, blunt trauma, vascular graft, autologous graft

## Abstract

Orthopedic procedures, although commonly associated with complications such as joint infections, deep venous thrombosis, or wound infections, rarely result in vascular complications. We present a case of a posterior tibial artery (PTA) pseudoaneurysm following blunt trauma during an elective orthopedic procedure involving the removal of osteosynthesis material. The patient developed leg swelling and intermittent hemorrhage between 1 and 3 weeks postoperatively. Doppler ultrasonography and contrast-enhanced computed tomography (CT) angiography confirmed the presence of a PTA pseudoaneurysm. The patient subsequently underwent open surgical repair in our secondary center, which involved exclusion of the pseudoaneurysm at the proximal PTA and patch angioplasty using an autologous venous graft. This case highlights the critical role of non-invasive diagnostic modalities, such as Doppler ultrasonography and contrast-enhanced CT with 3D reconstruction, in diagnosing vascular complications following orthopedic procedures. The patient remained asymptomatic during both the 3-week and 3-month follow-up periods.

## Introduction

Arterial complications, such as pseudoaneurysms, are infrequently documented following orthopedic procedures [1–3]. However, they can lead to significant morbidity and pose a threat to limb viability, particularly among elderly patients. Vascular injuries associated with elective orthopedic surgeries are more prevalent during revision procedures and in individuals with pre-existing atherosclerotic conditions.

## Case report

**Figure 1:**
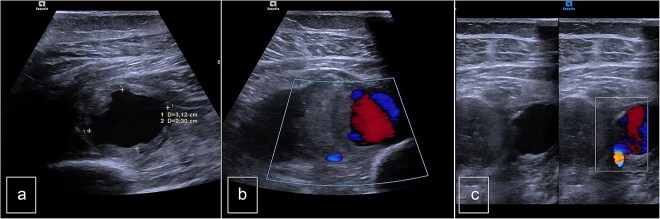
Preoperative duplex sonography. (a) M-mode. Pseudoaneurysm of the posterior tibial artery (vascularized anechogenic pocket). (b) Doppler mode and (c) Doppler mode. Identification of communication with the medial wall of the PTA.

The patient was a 55-year-old female who was admitted to the Surgery Department due to recurrent bleeding and swelling at the surgical site following the removal of elective osteosynthesis material from the left tibia and patella by orthopedic surgeons. Initial investigation involved Doppler ultrasonography of the left leg, which revealed a large hematoma within the deep muscle structures of the proximal and middle thirds of the calf. An anechogenic pocket, exhibiting arterial flow on Doppler-color imaging, was identified, consistent with a pseudoaneurysm with dimensions of 28 × 20 × 24 mm, and it was supplied by arterial flow from the posterior tibial artery (PTA). The PTA remained patent distally but demonstrated a demodulated, monophasic flow with a velocity of 12 cm/s (Fig. 1).

**Figure 2:**
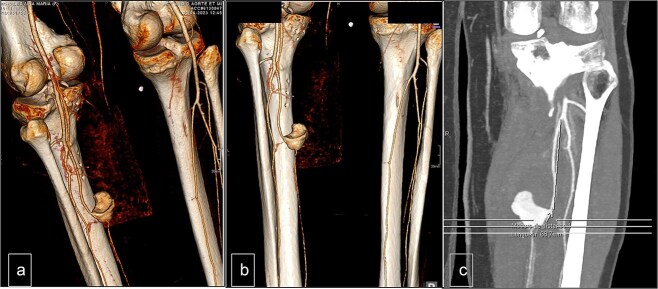
Computed tomography. (a) Lateral view, (b) posterior view, and (c) vascular reconstruction. The medial wall of the posterior tibial artery appeared discontinuous over a length of ~6 mm.

The Doppler ultrasonography was supplemented with contrast-enhanced computed tomography (CT angiography) and 3D reconstructions. The CT scan identified a hematoma with a pseudoaneurysm located at the inferior aspect, measuring 28 mm in height, 29 mm in the transverse axis, and 20 mm in the anteroposterior axis. The lesion was supplied by the PTA, which flowed laterally to the pseudoaneurysm. The medial wall of the PTA appeared discontinuous over a length of ~6 mm (Fig. 2).

**Figure 3:**
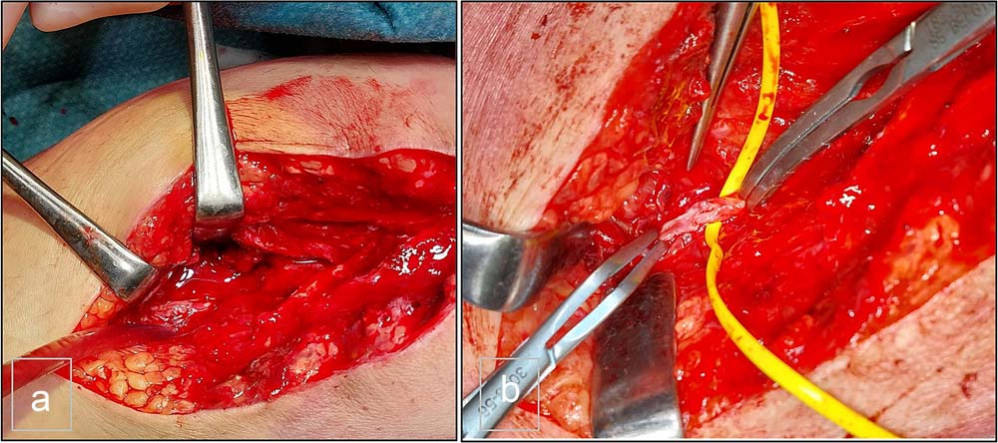
(a) Preoperative view. (b) Preoperative view. PTA after resection of the pseudoaneurysm and before the autologous patchoplasty.

Surgical intervention was performed, consisting of open resection of the pseudoaneurysm at the proximal portion of the PTA, followed by patchoplasty with an autologous vein graft from the homolateral limb. The posterior tibial vein was ligated during the procedure (Fig. 3).

**Figure 4:**
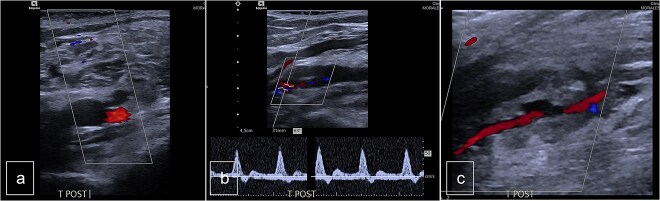
Postoperative duplex sonography. (a) Doppler mode. Identification of the PTA. (b) Doppler mode. Triphasic physiological flow in distality of the autologous patch. (c) Doppler mode. Permeability of the PTA.

The patient underwent follow-up assessments at 3 weeks and 3 months, which showed an uneventful recovery. Postoperative Doppler sonography demonstrated normal physiological flow (Fig. 4).

## Discussion

Arterial complications following orthopedic surgeries are relatively rare. Pseudoaneurysms of the below-the-knee arteries represent a potentially limb-threatening complication of procedures such as knee arthroplasty or osteosynthesis, often resulting from blunt trauma. These pseudoaneurysms can mimic the clinical presentation of deep vein thrombosis, with symptoms such as leg swelling, calf muscle pain, and impaired mobility, making diagnosis occasionally challenging.

In terms of pathophysiology, blunt trauma weakens the vascular wall, which may lead to the formation of a pseudoaneurysm. This may remain confined to a small muscular space or progress to more severe complications, such as rupture, infection, or even the development of a deep arteriovenous fistula.

Recurrent bleeding at the surgical site after orthopedic procedures should raise suspicion for the development of a pseudoaneurysm. In some cases, leg swelling and pain may be the only early warning signs.

Non-invasive vascular assessment methods, including Doppler ultrasonography and contrast-enhanced CT with 3D reconstructions, are valuable tools for diagnosis. Intraoperative angiography via femoral puncture may also be performed to assess the anatomy of the pseudoaneurysm and the vascular status of the affected artery.

Various treatment strategies have been described for managing pseudoaneurysms in the literature. These include thrombin injections, endovascular techniques such as coil embolization, and ligation of the feeding vessel.

In this case, we opted for open surgery and direct surgical repair of the PTA. This approach was supported by several considerations: firstly, the preoperative imaging confirmed vascular patency, and secondly, intraoperative backflow from the distal portion of the PTA was observed. The patient’s young age was also a factor in favoring vascular preservation. In older or more fragile patients, direct ligation of one of the three vessels below the knee may be considered when arterial preservation is not feasible.

To our knowledge, there are no reports in the literature documenting pseudoaneurysms of the PTA. However, a few case reports have described pseudoaneurysms of the anterior tibial artery [2] or popliteal artery [1, 3] following elective orthopedic surgeries [4, 5]. Injuries to the PTA are less common than those to the popliteal or anterior tibial arteries, likely due to anatomical factors and its location in the medial aspect of the tibial diaphysis. Endovascular stenting [6] or coil embolization [7] may serve as an alternative treatment option in highly selected cases. Early diagnosis and timely intervention are crucial for achieving favorable outcomes in these patients [8].
